# Evolution of the COVID-19 Pandemic after the Introduction of School Referral Nurses in the Province of Granada—A Descriptive Study

**DOI:** 10.3390/children9111646

**Published:** 2022-10-28

**Authors:** Juan Carlos Sánchez-García, Ana Eugenia Marín-Jiménez, María Isabel Tovar-Gálvez, Jonathan Cortés-Martín, María Montiel-Troya, María José Menor-Rodríguez, Raquel Rodríguez-Blanque

**Affiliations:** 1 Andalusian Plan for Research, Development and Innovation, CTS-1068, 18014 Granada, Spain; 2 Department of Nursing, Faculty of Health Sciences, University of Granada, 18016 Granada, Spain; 3 Quantitative Methods for the Economics and Enterprise, Faculty of Economics and Business Sciences, University of Granada, 18071 Granada, Spain; 4 Department of Nursing Ceuta Campus, Faculty of Health Sciences, University of Granada, 51001 Ceuta, Spain; 5 Deputy Director of Humanization and Citizen Services, Santiago-Barbanza Health Area, 15706 Santiago de Compostela, Spain; 6 San Cecilio University Clinical Hospital, 18071 Granada, Spain

**Keywords:** health behaviour, children, COVID-19, nursing scholar, prevention, schools

## Abstract

The aim of this research is to describe the evolution of the pandemic in a school context, following the introduction of school nurses into the educational setting. Background: The first wave of COVID-19 in Spain prevented social interaction by imposing lockdowns on the population. All non-essential activities, including face-to-face education, were interrupted, affecting the school-aged population during the second and third trimester of the 2019/2020 school year. Schools are places where prevention, identification and case management measures should be rapidly implemented. Methods: This is a prospective and descriptive study using a quantitative method to study the school population of Granada and its province during the school year 2020/2021, from September 2020 to May 2021. The study participants were all schools under the jurisdiction of the Territorial Delegation of Education of Granada, whether public, private, state-subsidised or charter schools, including all educational stages. Results: The confirmation rate in Granada city (11.2%), in contrast to the Andalusian average (6.9%), places Granada as the province with the highest incidence rate. The infection rates among teachers show the same confirmation rate as the general population of Granada (9%); however, among students this rate is lower (7%). There is a higher incidence of outbreaks taking place outside school and a lower incidence of outbreaks occurring within the school environment. Both partial and total outbreaks are more frequent in early childhood education. Conclusion: The early identification and management of reported suspected cases of COVID-19 in schools is proving effective in preventing infection in the school population, achieving good pandemic control in schools, and reducing the number of outbreaks and people affected. Schools have been confirmed to be safe. Establishing screening for asymptomatic schoolchildren could be a tool to improve control in schools.

## 1. Introduction

We are currently experiencing a pandemic caused by SARS-CoV-2, which leads to a disease called COVID-19, which started in the Chinese city of Wuhan in December 2019. 

In Spain, as of 31 May 2021, more than 3,687,762 confirmed cases have been registered, of which 80,049 have resulted in death, according to data provided by the Ministry of Health, Consumer Affairs and Social Welfare [1].

The first wave in Spain was approached by avoiding social interactions through the lockdown of the population and suspending all non-essential activity, including face-to-face teaching activity, affecting the complete school-age population during the second and third trimester of the 2019/2020 academic year.

Education is a right, and has a fundamental role in the development of individuals and of society, so the interruption of educational activity has been related to increased inequality, and is not justified as an effective strategy to confront the new coronavirus [2]. According to the authors of the Malala Foundation report *Reopening schools during the COVID-19 pandemic*, the reopening of schools should be a priority over the reopening of other components of society, as the closure of schools will impact on educational enrolment rates and increase inequalities in the long run [3]. 

Europe started the school year earlier than Spain, and there were many reports of transmission inside classrooms and complete school closures. In Spain, there was a clear commitment to face-to-face education, with prior planning for a “safe” return to the classroom, and plans were designed and implemented for the adaptation of educational centres, based on guidelines issued by the national government. In Andalusia, the Andalusian Ministry of Health and Families created a network of nurses for specific assistance in cases of COVID-19 in the school environment. 

Educational centres are places where there are multiple interactions between the people who gather there, and in certain age groups it is not possible to maintain safety measures, leading to circumstances that may facilitate the transmission of the virus. It is essential to quickly carry out prevention, identification and case management measures.

The closure of schools has negative repercussions in both the short and medium term, negatively influencing both the socialisation of children and the acquisition of knowledge that ultimately improves the health status of populations. It has also been linked to the increase in already identified inequality gaps, and fewer opportunities for families with more limited economic and technological resources [4]. 

Studies on children and young people in relation to mental health disorders caused by the pandemic and lockdown show an increase of up to 20% in anxiety disorders and depression, with these figures being higher in areas identified as the epicentre of the pandemic [5].

Considering all the negative impacts identified, a return to face-to-face teaching was planned for the 2020–2021 school year, with the intention of offering a safe environment for both schoolchildren and teaching and support staff.

Effective pandemic control requires adherence to a combination of measures concerning both individuals and the environment, which can be categorised as personal hygiene, contact limitation, cleaning and ventilation, and case management. It is at this point in the management of cases that a healthcare professional role, dedicated exclusively to the management of cases of COVID-19 in educational centres, the COVID-19 reference nurse (CRN), is created.

The CRN for educational centres works with total dedication to the management of COVID-19 cases that affect schoolchildren. 

The province of Granada is divided into four health districts, each with a district reference nurse (DRN) and a number of school nurses (SN) according to the number of schools covered by each district: Granada (13 SN), Metropolitan (16 SN), South Health Management Area (H.M.A.S.) (6 SN) and the Northeast Health Management Area (H.M.A.N.E.) (4 SN).

Therefore, it is of interest to analyse the evolution of the pandemic in a school context after the introduction of school nurses into the educational environment during the school year 2020/2021. This study includes a comparison between the incidence of cases of COVID-19 in the province of Granada and in the Andalusian community (distinguishing by sex), as well as the incidence of cases of COVID-19 in the school environment in comparison with the general population. We aimed to determine the incidence of the disease among students and teachers to determine the number of outbreaks in the school environment and the relationship with the population, and to study the rate of cessation of activity in the educational environment after the implementation of school nurses in the educational environment.

## 2. Materials and Methods

Because de-identified data from public sources were used, this cohort study was considered to be exempt from institutional review board approval and informed consent from patients was not required. This study is in accordance with the Strengthening the Reporting of Observational Studies in Epidemiology (STROBE) reporting guidelines.

### 2.1. Study Design

This was a prospective, descriptive study that utilised a quantitative method.

### 2.2. Setting and Participants

The study was carried out in the school population, considering both schoolchildren and teachers in the province of Granada. All schools belonging to the Territorial Delegation of Education in Granada participated in the study, regardless of their funding: public, private, state-subsidised or charter schools, including different educational stages, were all involved.

Data collected in the 2020/2021 school year, from September 2020 to May 2021, were studied.

### 2.3. Variables

- Sociodemographics: The student’s age and academic level were recorded.
- Incidence of COVID-19 in the school environment: Incidence rates of COVID-19 were recorded among the student body and among teaching staff and auxiliary and service staff (ASS).
- Number of suspected cases reported in the school setting registered through the SENECA platform (suspected students—Est SUSP; suspected teachers—Teac SUSP; and auxiliary and service staff—ASS SUSP); number of confirmed cases (confirmed students—Est CONF; confirmed teachers—Teac CONF; and confirmed auxiliary and service staff—ASS CONF) detected by reviewing the results of diagnostic tests for active infection (DTAI) including PCR (polymerase chain reaction) and antigen tests performed in the public and private healthcare system of Granada; and number of close contacts (Cont ESTR) associated with each confirmed case, declared through Annex III (official document of communication to the public health services, where the number of close contacts associated with a confirmed case are collected).
- Number of classrooms with partial cessation of teaching activity due to the detection of COVID-19 cases that met the epidemiological criteria for the cessation of activity, expressed in quarters.
- Number of centres with total cessation of teaching activity as a consequence of the occurrence of COVID-19 cases, meeting the epidemiological criteria for total cessation of activity, expressed in quarters.
- Number of school and non-school outbreaks registered in the Alert Network.

### 2.4. Data Sources/Measures

The information was collected from the following registers:

- IECA Portal: The collaboration of the Andalusian Institute of Statistics and Cartography with the Regional Ministry makes the information on the evolution and behaviour of the pandemic visually accessible and allows the reuse of these data by users, contributing to its accessibility and transparency.
- School Apex and Tracer Apex platforms, under the Ministry of Health and Families of the Andalusian Regional Government, created ad hoc for the registration and tracing of suspected and confirmed cases, as well as close contacts of COVID-19.
- SENECA portal of the Andalusian Regional Ministry of Education and Sport: the COVID-19 care registration version, developed by the Regional Ministry ad hoc for COVID-19 case management.
- University Hospitals “Hospital Clínico San Cecilio” and “Virgen de las Nieves”: Daily report of the results obtained in the microbiology laboratories of the hospitals with regard to tests for COVID-19.
- DIRAYA: Daily notification of rapid antigen tests carried out in primary care and declared through the DIRAYA PDIA register (healthcare information system of the Andalusian Health Service (AHS) which consists of the integration into the users’ single health record and contains all the citizen’s healthcare episodes registered by AHS professionals).
- Alert Network: Network dependent on the Andalusian Public Health System that registers situations of risk to public health.

### 2.5. Bias

The researchers have not intervened by modifying data from their perspective. Data were extracted from public primary sources of the Spanish Government and the Junta de Andalucía.

In order to avoid misleading data, positive or negative results from self-testing were discarded and only PCR and antigen tests performed in official laboratories were considered.

### 2.6. Sample Size

Data were selected from all students and teachers in the educational centres of the autonomous community of Andalusia. 

### 2.7. Quantitative Variables

Different types of education were defined; preschool education cycles (1st and 2nd cycle), primary education, compulsory secondary education, baccalaureate/training cycles, special education (specific special education classrooms, integration support classrooms, artistic education, music, dance, theatre, languages and sports education) and adult education (classroom-based secondary education, distance learning, preparation for secondary education access examination). 

On the other hand, four “waves” or periods were defined: “1st wave” or Period 1 (from the beginning of the epidemic until 10 May 2020), “2nd wave” or Period 2 (from 11 May 2020 to 20 December 2020 inclusive), “3rd wave” or Period 3 (from 21 December 2020 to 7 March 2021 inclusive) and “4th wave” or Period 4 (from 8 March to 31 May 2021).

### 2.8. Statistical Methods

The quantitative data collected were summarized using descriptive statistics and are presented using frequency tables and percentages. The data analysis performed included the Mann–Whitney U test or the Kruskal–Wallis test depending on whether two or more groups were being compared. The margin of error or confidence interval was set at 95% with a standard deviation of 0.05. A two-factor test such as the ANOVA test could not be performed because the public health administration did not provide the raw data.

All statistical analyses were carried out with the IBM SPSS 23 statistical software (SPSS Inc., Chicago, IL, USA).

## 3. Results

In the province of Granada, according to the data provided by the Territorial Delegation of Education, there are 876 educational centres with 8760 classrooms, including a total of 205.350 students, distributed as shown in Table 1.

Andalusia has a population of 8,464,411. According to data from the IECA portal, since the beginning of the pandemic, 1,402,083 confirmed cases of COVID-19 have been counted. 

In the autonomous community of Andalusia, all provinces have been affected by the new coronavirus, with the highest number of cases in the province of Seville with a total of 128,239 cases (1,957,688 population) and a confirmation rate of 6551/100,000 inhabitants, followed by Malaga with 101,102 (1,685,920) and a confirmation rate of 5997/100,000 inhabitants, and Granada with a total of 87,787 (919,168) and a confirmation rate of 9,551/100,000 inhabitants.

During the “1st wave”, the province with the highest number of confirmed cases was Malaga (4080), followed by Granada (3087) with a difference of 1000 cases; in the “2nd wave”, the province with the highest number of confirmed cases was Seville with 56,370 cases, followed by Granada with 43,659 cases. In the “3rd wave”, Malaga was once again the province with the highest number of confirmed cases, with 49,281 cases followed by Seville with 43,418, and in the “4th wave” the province with the highest number of cases was Seville with 25,472 cases, followed by Granada with 17,320 cases.

The distribution of confirmed COVID-19 cases by age in the autonomous community of Andalusia and within the province of Granada can be seen in Table 2.

**Figure 1:**
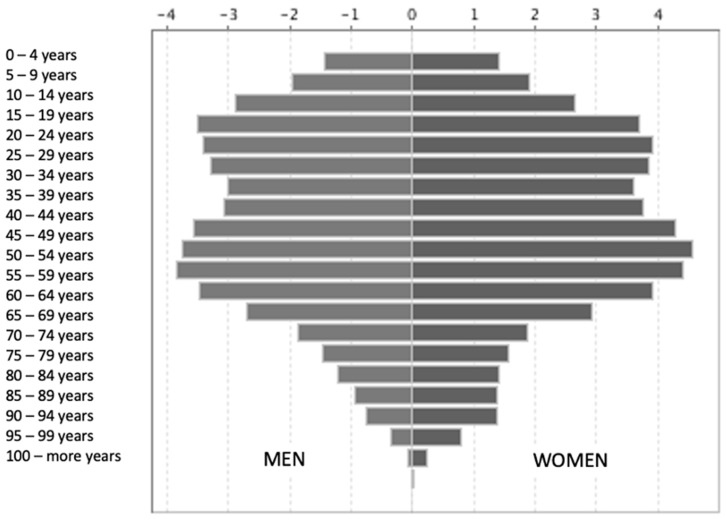
Incidence of COVID-19 in the province of Granada grouped by sex and age. Source: IECA portal on the COVID-19 in Andalusia.

We observed that, in Andalusia, there is a higher infection rate among women than in men, in all age groups (Figure 1), except in the group of 0- to 14-year-olds, where infection rate is higher among males. The same pattern is found in the province of Granada, where in the age group for 85 years and above, the number of infections in women is more than double the infections among men (2117 women vs. 1017 men) and the age groups where the highest number of cases are found are the groups between 15 and 29 years of age, and groups between 40 and 59 years of age.

**Figure 2:**
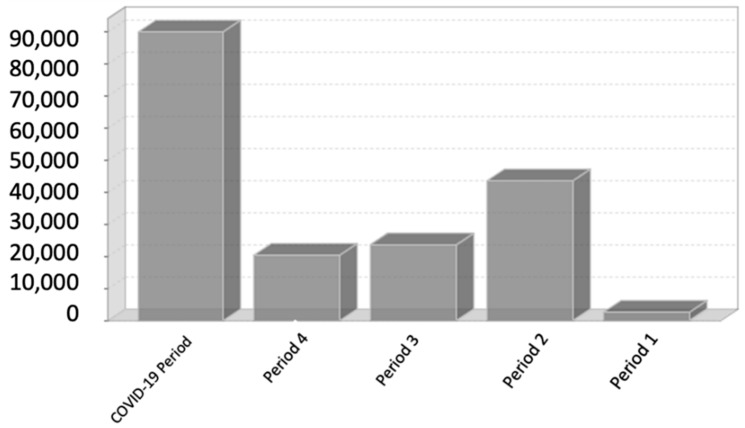
Evolution in the province of Granada by periods. Source: IECA portal on COVID-19 in Andalusia.

Figure 2 shows the evolution of the pandemic in the province of Granada by periods. 

The pandemic has had an unequal impact on the population depending on the health measures implemented.

Regarding the results obtained on the incidence of the disease among students and teaching staff during the 2020/2021 school year, we found an incidence rate of 9% among teachers/staff, with a total of 1565 teachers confirmed. Among students, there was a total of 15,478 students (7%). 

The results of studying outbreaks among the school population and the general population are shown in Table 3.

When considering the different outbreaks, distinguishing between school and non-school outbreaks, and the different periods, we found that there was a statistically significant difference *p* = 0.004, with a lower value among school outbreaks compared to non-school outbreaks.

In the period from September to 20 December, where students were allowed to return to their classrooms, 301 non-school outbreaks were detected (84.1% in the period) compared to 57 school outbreaks (15.9% in the period). It was during this period that the highest percentage of both types of outbreaks took place (68.7% of non-school outbreaks and 52.3% school outbreaks). During Period 3, there were 75 non-school outbreaks (70.1% in the period) and 34 school outbreaks, whereas in Period 4 there were 62 non-school outbreaks (75.6% in the period) and 20 school outbreaks (24.4% in the period).

The evolution of the partial and total cessation of classroom teaching activity has been closely related to the incidence of the pandemic in the general population, and very closely related to the way classes are organized and the relationships between schoolchildren, with cessation occurring more frequently in preschool education cycles, as they are organized as stable coexistence groups.

**Figure 3:**
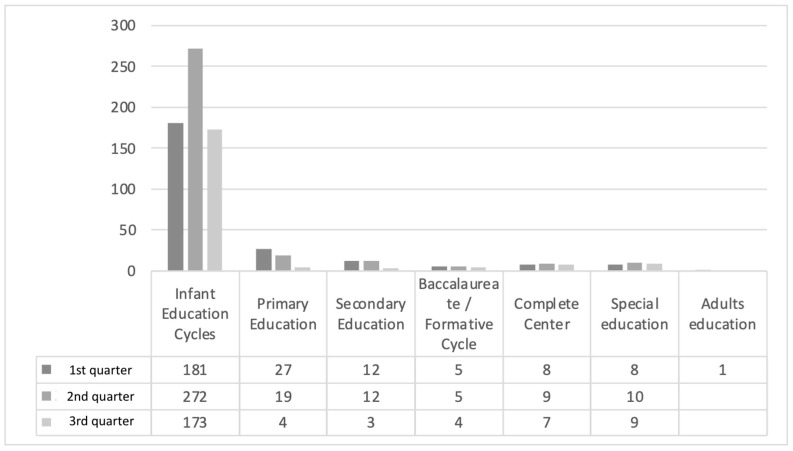
Classrooms closed during the school year by training cycle *. Source: Own elaboration. * *p* = 0.006.

With regard to the closure of classrooms per quarter in relation to education cycle, we obtained a statistically significant value (*p* = 0.006) via the chi-square test. During the second quarter, there was a higher number of closed classrooms with 327 classrooms (42.5%), followed by the first quarter with 242 closed classrooms (31.5%), and finally the third quarter with 200 closed classrooms (26%). The results for closed classrooms according to the formative cycle can be found in Figure 3. 

## 4. Discussion

In this study, we observed that the city of Granada has a higher confirmation rate (11.2%) than the Andalusian average (6.9%). This higher incidence of the disease in the province of Granada has not been scientifically explained, and many hypotheses have been proposed by experts to justify these figures. Among them, one suggestion is related to the higher population density in an acute form and the movement of people as a university city. There is also a sector that is decreasing as a result of the higher number of DTAI tests carried out since the start of the pandemic, which has made it possible to detect a larger number of positive cases of COVID-19 disease. 

Regarding the total or partial cessation of face-to-face education, this is based on epidemiological criteria related to the incidence of outbreaks and transmission within the classroom. There are countries such as Israel where, after opening schools on 1 September 2020, they had to return to a total closure of schools on 14 September, reopening them on 1 November [6]. However, in Andalusia, with an infection rate in the second period among the highest in the world, there was no complete closure of schools, which may have been a result of the school nurse management model in practice.

The end of the second wave of the epidemic saw the highest number of total and partial school closures. This may be explained by the initial difficulty in implementing the COVID-19 monitoring protocol in schools, where the reference criteria for the cessation of classroom activity were not well defined, as there was no previous information available in schools, as well as the higher percentage of truancy, especially identified in early childhood education cycles, which responded to the lack of confidence of parents in their children’s attendance to schools and the voluntary nature of education at this stage [7]. Both partial and total truancy were more frequent in preschool education cycles, since prevention measures cannot be maintained among younger children; however, the study of subsequent contacts, in many cases, corroborates what different authors show in their articles, associating a lower capacity for transmission of the virus at earlier ages [8].

The distribution of confirmed cases in schools in the province of Granada has not followed the same pattern as in the general population. By health districts, the metropolitan district has the highest rate of confirmed cases in schools, and the southern health management area has the lowest. The fact that the coastal area has a lower rate of confirmation among pupils and the general population may be related, according to some experts, to the negative association between the relative humidity of the environment and the lower incidence of COVID-19 [9].

Infection figures among teachers reflect the same data as the general population in Granada. However, among students, this figure is lower, a fact that may be related to the lower number of social relationships that take place among children, and to the rapid management of suspected and confirmed cases thanks to the nursing management model, which allows the immediate quarantine or isolation of contacts and cases, thus avoiding transmission among other schoolchildren [8]. Data from this study show that the percentage of students who have been infected with COVID-19 during the period between the start of the school year (10 September) and 31 May is 7%, or 15,478 students. This rate is higher among teaching staff (9%), amounting to 1565 teachers/staff. This was not the situation in the study by Mensah et al. [10]. There, they describe that, in school-aged children, SARS-CoV-2 infections followed the same pattern as adult cases and only declined after national containment was implemented, keeping schools open. In the study by Somekh et al. [6], after exploring the impact of the spread of SARS-CoV-2 in the population aged 0–19 years in schools in Israel, they report that the results suggest that children aged 0–9 years did not have significant rates of SARS-CoV-2 infection during periods of school attendance.

The evolution of the SARS-CoV-SA2 pandemic in schools mirrors the evolution of the pandemic in the community context, according to Li et al. [11], as children’s interactions take place not only in the school environment, but also in contexts such as family and leisure time in other out-of-school situations. The school could be a potential source of transmission between pupils and school workers [12], a fact that was not found in our study, as we found that the highest number of outbreaks occurred outside the school and that fewer people were affected in outbreaks occurring within the school environment. 

School lockdown is not justified, except as an extreme measure in situations of uncontrolled community transmission, and should be combined with other prevention measures involving all sectors of the population [13]. There is no significant association between face-to-face education and COVID-19 incidence rates, as demonstrated by a study carried out by professors at Binghamton University in New York (USA) [14], suggesting that it does not make sense to break up students’ learning experiences. 

One of the most common facts is the difficulty of collecting official data on the impact of the pandemic on the population.

Therefore, this study has been very limited in finding official published data on the incidence of COVID-19 in the general population, and this limitation is even more important when studying the impact of COVID-19 in the school population. There are no scientific articles currently available that reflect data on the incidence of COVID-19 in schoolchildren, teachers or support staff. There are also no studies reporting on the relationship between school outbreaks and outbreaks in the general population.

Our work has collected the data provided by different official sources of information described in the methodology, as well as the data provided by the platforms specifically created for the management of COVID-19 cases in the educational environment.

As a bias, because of the ad hoc programmes created for the control of the pandemic and considering the important incorporation of professionals within the Territorial Delegation of Education and Sport of Granada, there may have been an under-reporting of cases by the COVID-19 coordinator of the educational centre. As a mechanism to control the possible under-reporting of cases, the number of cases confirmed as positive, issued by the reference microbiology laboratories in Granada and the province, as well as the cases declassified in the Alert Network of the Andalusian public health system, specifically in the province of Granada, were reviewed daily.

Finally, this research shows the efficacy of school nurses dedicated exclusively to pandemic control. The early detection and rapid management of confirmed and suspected cases of COVID-19 have been shown to be effective measures for pandemic control in the educational setting. Having a school nurse in schools increases the safety of school children in the classroom, supporting face-to-face education in a pandemic environment.

## 5. Conclusions

The introduction of COVID-19 nurse case managers into schools has facilitated the rapid management of suspected and confirmed cases and identifying close contacts, and is proving effective in preventing infection among the school population.

Schools have been confirmed as safe centres, where there is no higher risk of infection than in other environments and which have not been a source of the spread of infection.

Prevention measures put in place by health authorities, together with a fast case management response, are crucial to achieve good pandemic control in schools and reduce the number of school outbreaks.

The suspension of face-to-face teaching activity happens more frequently in preschool and special education because of the special characteristics of these groups.

Schools should remain open for face-to-face learning without substantially increasing community SARS-CoV-2 case rates.

## Figures and Tables

**Table 1:** Distribution of students in 2020–2021 school year, Granada province (prepared by the authors).

Type of Education		Number of Students
Preschool Education Cycles	1st CYCLE	10,192
	2nd CYCLE	25,008
Primary Education		55,982
Obligatory Secondary Education		42,457
Baccalaureate		13,144
Formative Cycles		18,766
Special Regime		11,301
Adult Education		25,571
**TOTAL STUDENTS**		205,350

**Table 2:** Confirmed cases of COVID-19 in Andalusia and Granada province. Source: IECA portal on COVID-19 in Andalusia. Prepared by the authors.

Place of Residence	Age	Sex	Total Confirmed	Rate × 100.000 Inhab.
Andalusia	TOTAL	Both sexes	585,768	5.86
		Male	277,224	2.77
		Female	308,511	3.09
Granada	TOTAL	Both sexes	87,787	0.88
		Male	40,841	0.41
		Female	46,944	0.47

**Table 3:** Relationship between type of outbreaks and periods (own elaboration). *p* = 0.004.

			Period			Total
			September to 20 December	3rd	4th	
**Type of outbreak**	Non-school	Count	301	75		438
		% within type of outbreak	68.7%	17.1%	14.2%	100%
		% within period	84.1%	70.1%	75.6%	80.1%
	School	Count				109
		% within type of outbreak	52.3%	29.4%	18.3%	100%
		% within period	15.9%	29.9%	24.4%	19.9%
Total		Count	358	107	82	547
		% within type of outbreak	65.4%	19.6%	15%	100%
		% within period	100%	100%	100%	100%

## Data Availability

Available upon request from the corresponding author.
